# The accuracy and usability of point-of-use fluoride biosensors in rural Kenya

**DOI:** 10.1038/s41545-023-00221-5

**Published:** 2023-02-08

**Authors:** Walter Thavarajah, Patrick Mbullo Owuor, Diana Ross Awuor, Karlmax Kiprotich, Rahul Aggarwal, Julius B. Lucks, Sera L. Young

**Affiliations:** 1 Department of Chemical and Biological Engineering, Northwestern University, 2145 Sheridan Road, Evanston, IL 60208 USA; 2 Center for Synthetic Biology, Northwestern University, 2145 Sheridan Road, Evanston, IL 60208 USA; 3 Center for Water Research, Northwestern University, 2145 Sheridan Road, Evanston, IL 60208 USA; 4 Center for Engineering, Sustainability and Resilience, Northwestern University, 2145 Sheridan Road, Evanston, IL 60208 USA; 5 Department of Anthropology, Northwestern University, 1810 Hinman Avenue, Evanston, IL 60208 USA; 6 Institute for Policy Research, Northwestern University, 2040 Sheridan Road, Evanston, IL 60208 USA; 7 Program of African Studies, Northwestern University, 620 Library Pl, Evanston, IL 60208 USA; 8 Department of Management Science and Project Planning, Nairobi University, P.O. Box 30197, GPO, Nairobi, Kenya; 9 Department of Epidemiology and Medical Statistics, School of Public Health, Moi University, P.O. Box 4606-30100, Eldoret, Kenya

**Keywords:** Water resources, Developing world, Chemical engineering

## Abstract

Geogenic fluoride contaminates the water of tens of millions of people. However, many are unaware of the fluoride content due in part to shortcomings of detection methods. Biosensor tests are a relatively new approach to water quality testing that address many of these shortcomings but have never been tested by non-experts in a “real-world” setting. We therefore sought to assess the accuracy and usability of a point-of-use fluoride biosensor using surveys and field tests in Nakuru County, Kenya. Biosensor tests accurately classified elevated fluoride (≥1.5 ppm) in 89.5% of the 57 samples tested. Usability was also high; all participants were able to use the test and correctly interpreted all but one sample. These data suggest that biosensor tests can provide accurate, meaningful water quality data that help non-experts make decisions about the water they consume. Further scaling of these technologies could provide new approaches to track global progress towards Sustainable Development Goal 6.

## Introduction

Water contamination and its resultant health and economic burdens are a pressing global health concern^1^. Sustainable Development Goal (SDG) 6 tracks progress towards the “availability and sustainable management of water and sanitation for all”. Progress towards SDG target 6.1, the proportion of humans with “universal and equitable access to safe and affordable drinking water” is tracked primarily using data on drinking water infrastructure access reported by national statistics offices to the United Nation’s Children’s Emergency Fund (UNICEF) and the World Health Organization’s (WHO) Joint Monitoring Programme (JMP)^2^.

Current estimates based on JMP data indicate that two billion people worldwide lack access to safely managed drinking water service^2^, such that we are not on track to meet target 6.1 by 2030^3^. Even this estimate may be overly optimistic as current data on water quality are limited^4^. Specifically, less than half of the United Nations’ member states have the resources to generate water quality data robust enough to drive governance^3^. As such, there is an acknowledged need for more widely usable data collection technologies to track the presence of water contaminants identified by the WHO as priority^5^, specifically *E. Coli*, arsenic, nitrites, and fluoride^6^.

Dangerous levels of fluoride are found in water sources used by tens of millions of people worldwide^7,8^. Exposure to fluoride concentrations above 1.5 ppm (or 1.5 mg/L), the cut-off established by the WHO^6^, typically occurs when naturally occurring fluoridated salts leach into underground aquifers. Elevated levels of fluoride in groundwater occur globally, and is of particular concern in northern and eastern Africa, the Middle East, and parts of North and South America^9,10^. Although there are health benefits to fluoride exposure below 1 ppm, including prevention of dental caries^11^ and treatment of osteoporosis symptoms^12^, chronic exposure to high levels of fluoride has a number of adverse effects, most notably, dental and skeletal fluorosis^13^. Fluorosis embrittles teeth and bones by binding to the calcium within them, and can cause debilitating lifelong health complications^14,15^.

One of the biggest obstacles to mitigating exposure to harmful geogenic fluoride is the difficulty in identifying its presence: fluoride in water is colorless, odorless, and undetectable by taste below 2.4 ppm^16^. Fortunately, it is straightforward to accurately quantify fluoride levels in laboratory settings using techniques such as ion chromatography or ion-sensing electrodes^7^. Additionally, cutting-edge fluorescent probes capable of detecting nanomolar levels of analyte^17–19^ may offer an even simpler method for laboratory-based sample analysis. However, these technologies all require significant infrastructure and expertize to operate, necessitating a centralized approach to their use. A centralized approach, in turn, requires samples to be collected in the field and shipped to the laboratory, creating additional costs and logistical constraints in testing and communicating results in potentially affected areas.

Accurate point-of-use technologies currently exist to circumvent some of these limitations, but are of limited value to non-experts because of their cost, complexity, and/or accuracy^6^. For example, portable fluoride sensing electrodes and photometers can quantitatively measure fluoride levels in water onsite, but cost hundreds to thousands of dollars and require calibration procedures and maintenance for their use. Point-of-use chemical strips offer another field-friendly alternative that cost less than USD 1.00 per test, but are prone to false negatives and frequently fail to identify even extremely high levels of fluoride^20^. As such, there is a need for accurate, simple, and affordable methods that can be used by non-experts to accurately identify water sources with fluoride levels ≥1.5 ppm at the point of use. Such tests can both help people make decisions about the water they consume and track global progress towards SDG 6.

Cell-free biosensing technologies offer a promising strategy for the development of accurate, simple, and affordable water quality diagnostics^21^. Biosensors are naturally occurring RNA or protein systems in cells that sense compounds relevant to cell health. These natural systems work by binding interactions to the RNAs or proteins that then trigger the expression of genes that can in turn metabolize or export the compound. Synthetic biosensors can be created by extracting these natural systems out of the cell, and reconfiguring them to express genetically encoded reporter genes that lead to a visually detectable signal to indicate the target compound’s presence (i.e., color change). A key strength of these systems is that they operate as an in vitro reaction, outside of a living cell, and are therefore not genetically modified organisms. In addition, they can be freeze-dried and stored, facilitating manufacturing and transport to where they are needed. Rehydration of the tests with water samples thus allows them to be used as point-of-use water quality diagnostics. Furthermore, biosensing reagents cost on the order of tens of cents per test to produce (USD 0.73 for a test and a positive control)^22^, even in a laboratory (i.e., not at production scale). This makes them comparable favorably to the costs of gold-standard field-deployable technologies (USD 0.89, Supplementary Table 1).

**Fig. 1:**
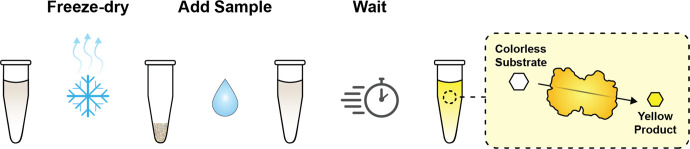
Schematic representation of a fluoride biosensor. A sensing reaction is prepared, freeze-dried, then rehydrated with a sample of interest. An enzymatic reaction occurs in the presence of fluoride, which converts a colorless substrate in the reaction to a yellow product.

For the detection of fluoride, a naturally occurring fluoride sensing mechanism from *Bacillus cereus* has been successfully engineered into a biosensor capable of detecting fluoride levels as low as 1 ppm and incorporated into a point-of-use fluoride test^20^. This test consists of a freeze-dried biosensing reaction that, when rehydrated with a water sample of interest, produces a visible yellow color in the presence of fluoride within hours (Fig. 1). This cell-free fluoride biosensor test was initially field-tested in a study in Cartago, Costa Rica^20^, a region with elevated levels of geogenic fluoride due to its proximity to the Irazu volcano, a known source of fluoridated salts^23^. In that study, tests were manufactured in Illinois and carried on board a commercial aircraft to the field site. Testing of nine different ground and surface water sources by a doctoral student revealed that the positive controls functioned in all cases, confirming that the basic biochemistry of the tests were robust to manufacturing, transportation, and field use. In addition, two samples were found to have detectable levels of fluoride. While promising, this study was limited by the small number of field samples tested, and more importantly, by the fact that tests were conducted by a single user with expertize in laboratory techniques and test operation. To assess usability, tests must be used by non-experts, and in a large enough sample size to calculate sensitivity and specificity.

We therefore explored the accuracy and usability of bioengineered point-of-use fluoride tests in Nakuru County, Kenya, a region with known geogenic fluoride contamination^24,25^. Specifically, we sought to evaluate test accuracy, assessed by the ability to correctly sense harmful levels of fluoride (established by the WHO as ≥1.5 ppm^6^) compared to photometry, a gold-standard method (Aim 1). We also tested usability, assessed by reported user experience with rehydrating and interpreting the tests (Aim 2).

## Results

### Study design and samples

We surveyed one member of each participating household to gather information about socio-demographics; drinking water sources; knowledge, attitudes, and behaviors about fluoride and fluorosis; and experiences with household water insecurity. We then characterized biosensor test accuracy by asking each participant to provide up to three household water sources and test them with the point-of-use biosensor. A second survey was conducted on the same day with the same participant to assess their experiences with using and interpreting the output of the biosensor test, and to ascertain and share fluoride concentrations obtained using a gold-standard method, i.e., fluoride photometer. Data collection is described graphically in Supplementary Fig. 1.

A total of 90 water samples were collected from 52 participants. Socio-demographics and knowledge, attitudes, and behaviors pertaining to fluoride and experiences with water insecurity were available for all 52 participants. The sample size available for evaluating test accuracy (Aim 1) and interpretation (Aim 2) was 57 water samples provided from 36 households. The number of samples was reduced from 90 to 57 because shipping conditions for the first batch of tests caused test degradation, making them unsuitable to evaluate accuracy and usability (see “*Test Kit Shipment to Nakuru County, Kenya*”).

### Socio-demographics and knowledge, attitudes, and behaviors of study participants concerning fluoride

The study included participants from a range of education and employment backgrounds, household sizes, and levels of water insecurity (Table 1). The majority of the 52 participants were women (73.1%), with a median age of 41 years. Roughly half of the participants had completed at least some secondary education. Participant occupations largely fell into three broad categories: agriculture, small businesses, e.g., market stands, or unemployed. Monthly household income ranged from KES 0–9500 (median USD 8.60). The median household size was 5 people; almost half of the households had children under five years old. Approximately one quarter of households were water insecure (HWISE score ≥12), i.e., they struggled with reliably accessing water to meet basic domestic needs.

**Table 1:** Sociodemographic characteristics of participants and their households in the point-of-use fluoride biosensor study in Nakuru, Kenya (*n* = 52).

Sociodemographic characteristics		Total households (*n* = 52)
Gender, *n* (%)		
	Female	38 (73.1%)
	Male	14 (26.9%)
Age (years)		
	Median (IQR)	41 (32, 50)
	Range	18–80
Education, *n* (%)		
	None	3 (5.8%)
	Some primary	11 (21.2%)
	Completed primary	10 (19.2%)
	Some secondary	8 (15.4%)
	Completed secondary	8 (15.4%)
	College/University	12 (23.1%)
Employment, *n* (%)		
	Agriculture	15 (28.9%)
	Small business	12 (23.1%)
	Employee	9 (17.3%)
	Unemployed	8 (15.4%)
	Unable to work	4 (7.7%)
	Student	2 (3.9%)
	Other	2 (3.9%)
Monthly household income		
	Mean	KES 1830 (USD 15.73)
	Median	KES 1000 (USD 8.60)
Total household size		
	Mean (SD)	4.9 (1.8)
	Median (IQR)	5 (4.6)
	Range	0–5
Number of children (≤15 years) in household		
	Mean (SD)	2 (1.43)
	Median (IQR)	2 (1,3)
Household Water Insecurity Experiences Score (0–36)		
	Mean (SD)	5.9 (8.9)
	Prevalence of water insecurity (≥12) *n*, (%)	14 (26.9%)

Most participants (73.1%) were knowledgeable about fluoride; they generally referred to it as a “salt” or “mineral” found in water. In addition, 7 participants mentioned that fluoride impairs dental and skeletal health, unprompted. When asked, most (90.4%) participants correctly identified some or all of the symptoms of fluorosis and the causal relationship between health problems and fluoride exposure. The majority of participants (71.2%) knew at least one person who had been affected by fluorosis.

This knowledge is contrasted by a comparative lack of understanding of how to take measures against fluoride exposure, with 42.3% of participants reporting that they didn’t know how to prevent fluorosis, and 34.6% reporting that they didn’t know how to treat it. Notably, while approximately half (48.1%) of participants correctly stated that using alternative sources of water and water treatment were methods to prevent fluorosis, fewer participants (26.9%) understood that fluorosis can only be treated with medical and dental care. The most commonly provided incorrect answer about fluorosis prevention and treatment was brushing teeth.

Although participants reported making efforts to avoid fluoride, fluorosis was not a major concern; 71.2% of participants reported that they never or rarely worried about fluorosis. Of the 33 participants (63.5%) who reported taking precautions against fluorosis, most (*n* = 27) reported using methods that were generally effective, including using water sources that were not known to be contaminated, diluting borehole water with rainwater, or treating their drinking water. However, 5 participants (9.6%) reported boiling their drinking water, which does not reduce fluoride content. Complete survey responses can be found under “Data Availability”.

### Characterization of biosensor accuracy

A total of 57 samples from 36 households were analyzed for test accuracy (Methods, Table 2). The majority of these water samples came from boreholes (49.1%), rainwater collection (19.3%), or protected dug wells (17.5%). The majority of provided samples (84.2%) were used for cooking, drinking, or both, but very few (7.0%) were treated to reduce fluoride. The water points were not located far from households; mean time to collect water was approximately 5 min, roundtrip.

**Table 2:** Characteristics of water samples available for assessment of accuracy of at-home biosensor fluoride tests (*n* = 57)^a^.

Characteristic		
Water sample source		
	Borehole/tube well	28 (49.1%)
	Rainwater	11 (19.3%)
	Protected dug well	10 (17.5%)
	Rainwater combined with borehole water	5 (8.7%)
	Surface water	1 (1.8%)
	Bottled water	1 (1.8%)
	Tap water	1 (1.8%)
Time needed for collection (roundtrip, in min)		
	Mean (SD)	5.4 (13.0)
Is sample used for cooking or drinking?		
	Yes	48 (84.2%)
	No	9 (15.8%)
Was sample treated?^b^		
	Yes	4 (7.0%)
	No	53 (93.0%)
Is respondent concerned about fluoride from this source?		
	Yes	10 (17.5%)
	No	47 (82.4%)
Fluoride concentration^c^		
	≥1.5 ppm	45 (78.9%)
	<1.5 ppm	12 (21.1%)

^**a**^The first 33 of the 90 water samples were not usable for assessment of accuracy because of biosensor test degradation due to shipment conditions.

^b^Treatment methods included chlorine tablets, distillation, and/or filtration.

^c^As ascertained by fluorimeter.

**Fig. 2:**
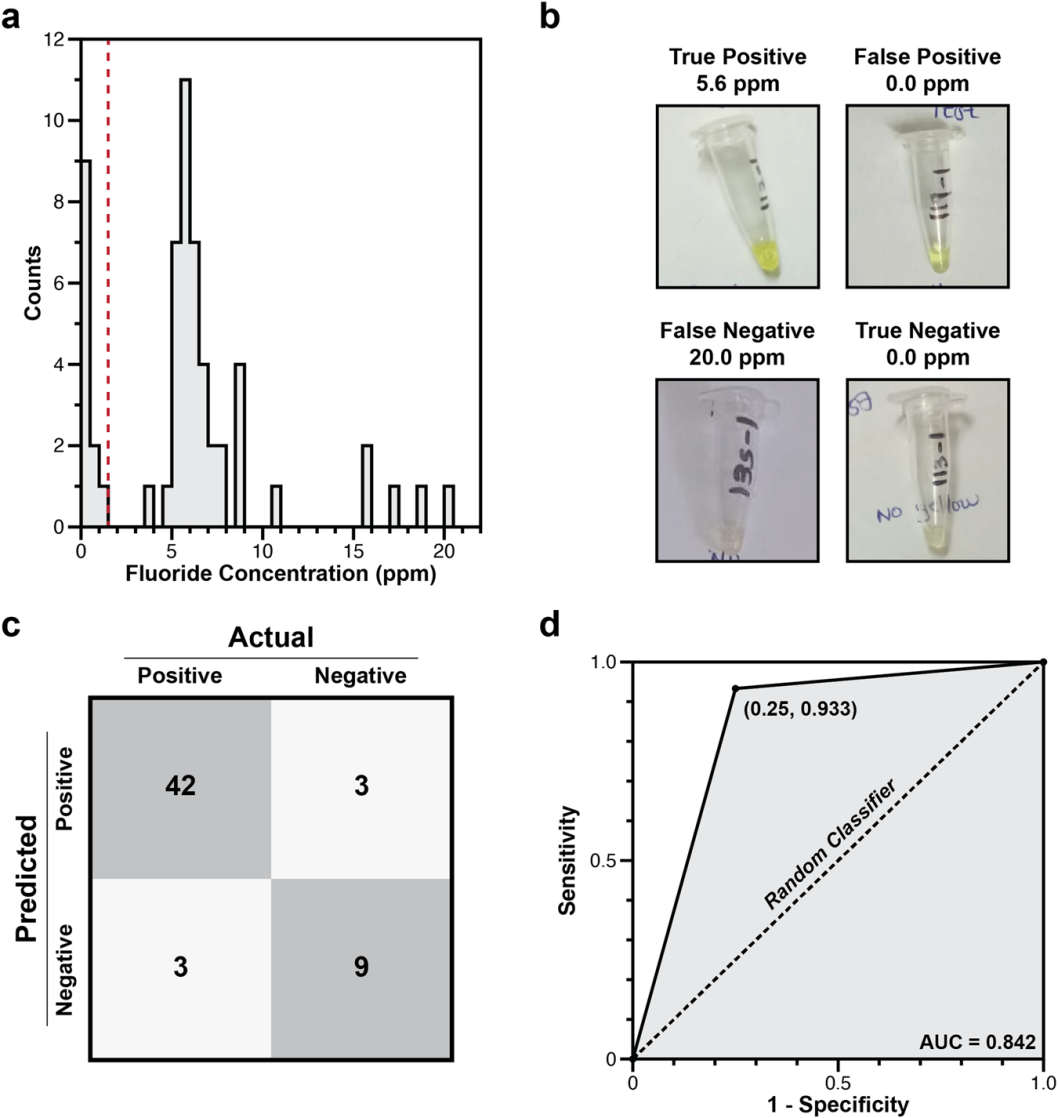
Fluoride content in 57 samples from 32 households, based on output from the point-of-use biosensor tests and the fluorimeter. **a** Distribution of fluoride concentrations in 57 water samples, as measured by fluorimeter. The red dashed line indicates the WHO guideline for elevated levels, ≥1.5 ppm. **b** Representative images of true positive, false positive, true negative, and false-negative test results. Photographs are annotated with fluoride concentrations measured by fluorimeter. **c** A confusion matrix of test results. “Actual” refers to classification by fluorimeter as being positive (≥1.5 ppm fluoride) or negative (<1.5 ppm fluoride). “Predicted” refers to biosensor test performance. “Negative” means no color change was observed, and “Positive” means a yellow color was visible. True positives and true negatives are shaded in gray, while false positives and false negatives are in white. **d** Receiver-operating characteristic curve derived from classifications in panel **c**. Sensitivity is calculated as (true positive)/(true positive + false negative) and specificity is calculated as (true negative)/(true negative + false positive).

Although participants were concerned about elevated levels of fluoride in only 10 of the 57 samples, fluorimeter analysis by field staff indicated that 45 had fluoride levels ≥1.5 ppm (78.9%), indicating a high prevalence of geogenic fluoride in drinking water (Table 2 and Fig. 2a). The measured fluoride levels were also high, with mean and median fluoride concentrations of 6.0 and 5.8 ppm, respectively. Most of the 12 uncontaminated samples were rainwater (83.3%), while most of the 45 contaminated sources were from boreholes (53.3%), protected dug wells (22.2%), or rainwater mixed with borehole water (11.1%) (Supplementary Table 2).

Six hours after the biosensor tests were rehydrated by study participants, field staff classified the output as positive for fluoride if a yellow color was observed, and negative for fluoride if no color change was observed. Comparison of these observations to the fluorimeter results allowed tests to be classified as true positive (yellow, with measured fluoride ≥1.5 ppm), false positive (yellow, measured fluoride <1.5 ppm), true negative (colorless, measured fluoride <1.5 ppm), false negative (colorless, measured fluoride ≥1.5 ppm) (Fig. 2b). Tabulating these results in a confusion matrix revealed that the biosensor tests correctly classified 51 samples (89.5%), and incorrectly classified 6 samples (10.5%) (Fig. 2c). The test sensitivity was therefore 93.3% (95% CI 81.7% to 98.6%) and specificity was 75.0% (95% CI 42.8% to 95.5%). Plotting these data on a receiver-operating curve revealed an area under the curve of 0.842 (Fig. 2d).

We identified no patterns among the incorrectly classified water samples in terms of water source or treatment. Furthermore, we observed that almost a fifth (*n* = 10, 17.5%) of the positive control reactions failed to activate (Supplementary Table 2). We did not observe any shared characteristics between the samples with failed positive controls. Furthermore, some true-positive tests had failed controls, indicating that the failure of the positive control for a given sample did not necessarily correlate to an incorrect classification by the test.

### Characterization of test usability

**Fig. 3:**
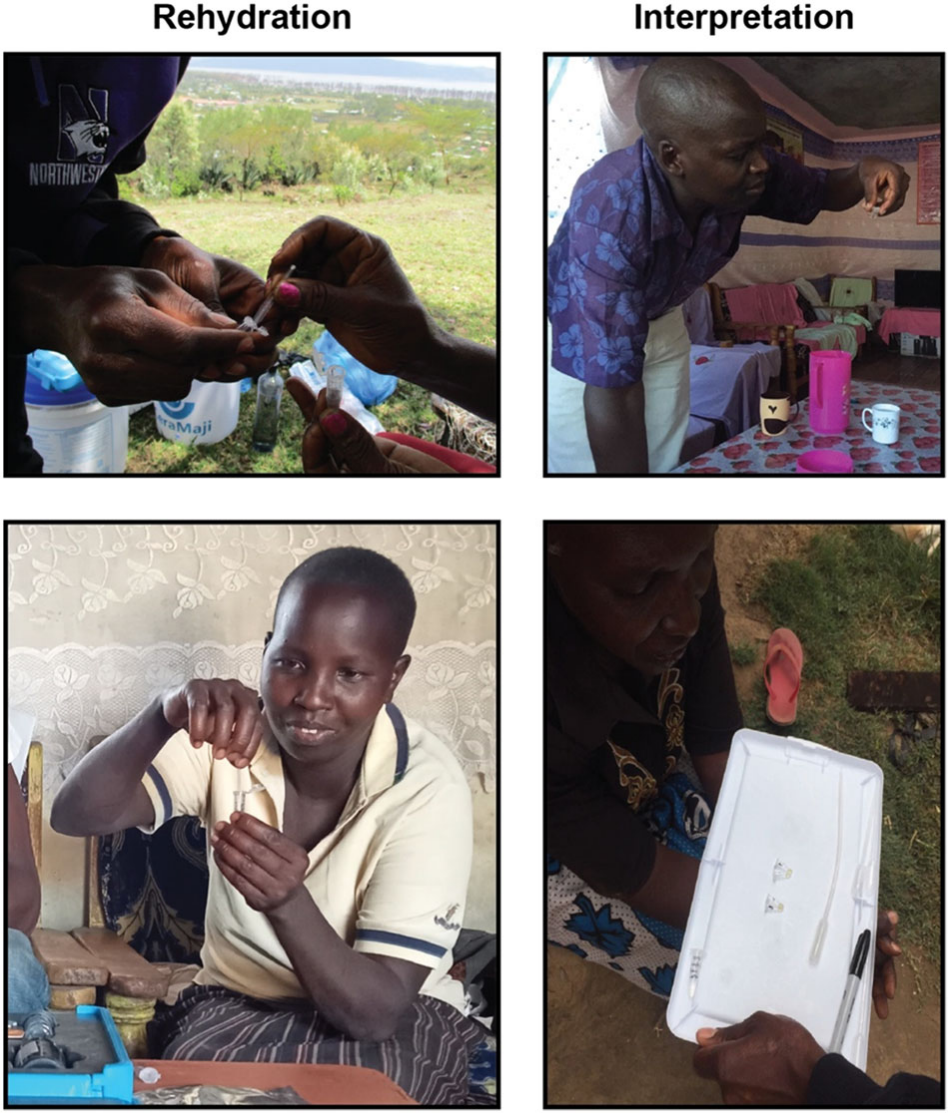
Representative photographs of experiences with point-of-use fluoride biosensor usability. The two key user activities for operating the tests are test rehydration, in which a micropipette is used to transfer a water sample into a microtubule (left) and result interpretation, in which the user ascertains if a yellow color has appeared (right).

To assess usability, we asked the 36 participants who provided water samples for accuracy (Aim 1) about their experiences with the rehydration and test interpretation of the tests. All participants were able to successfully transfer water into the PCR tube with a micropipette (Fig. 3, left), though two users (5.6%) experienced some difficulty dispensing the water. Owing to field constraints, especially the distance of participants’ houses from where field staff were staying, field staff were not able to be physically present with all participants to read the test results after 6 h, such that some participants were asked to assess if there was a color change before the reaction was complete (Fig. 3, right). At the time of readout, however, we observed agreement between participants and field staff in their assessments of the presence or absence of a yellow color in all but one of the 57 samples used for test interpretation assessment (98.2%) (Data Availability). There were no differences in usability by any sociodemographic characteristic, or by experiences with or knowledge, attitudes, and behaviors about fluorosis, or household water insecurity.

## Discussion

In what is, to our knowledge, the first description of field deployment and operation of any biosensor test by non-expert users, we found that a point-of-use fluoride biosensor test demonstrated a number of positive characteristics. To our first aim, it was accurate at detecting fluoride under field conditions, correctly classifying 89.5% of the 57 samples. Sensitivity was 93.3%, specificity was 75.0%, such that the area under the receiver-operating characteristic curve was .842, meaning that there is an 84.2% chance that the test will correctly predict fluoride contamination above the WHO limit of ≥1.5 ppm. Area under the curve values between 0.8 and 0.9 are generally considered “excellent”^26^.

To our second aim, these tests were highly usable. All participants were able to hydrate the tests, and there was only one test with a discrepancy between study staff and participant interpretation amongst the 57 samples used to assess test interpretation. In sum, participants were able to correctly identify public-health relevant concentrations of fluoride in their own household water sources, suggesting that the tests were eminently usable.

These tests fill a large unmet need for establishing the fluoride content of drinking water outside of a laboratory setting. Compared to the gold-standard laboratory methods, ion chromatography and ion-sensing electrodes, this biosensor enables fluoride testing without the need for resource-intensive infrastructure or trained personnel. Even compared to gold-standard point-of-use tests such as portable electrodes or the fluoride photometer used in this study, the biosensor has a simpler mode of operation and lower cost per sample tested. Indeed, at USD 0.73 per test (including a positive control) manufactured at a lab scale (Supplementary Table 1), this method is financially competitive with existing technologies; costs could be further reduced by scaleup.

Notably, these tests revealed a far higher prevalence of elevated fluoride levels than expected by participants. This suggests that such tests could reveal fluoride in other areas potentially affected by geogenic fluoride. They may also be useful in large-scale surveys of human health, well-being, and/or water security, such as those conducted by the World Bank, Gallup Poll, and the United States Agency for International Development. They could also be valuable in areas where the presence of fluoride is well-established, because of their ability to gauge water safety after measures are taken to remove fluoride. For example, the biosensor tests identified dangerous fluoride levels in the samples of borehole water even after it had been diluted with rainwater to reduce fluoride content.

The degradation of the first batch of tests clearly highlighted that the accuracy of point-of-use biosensors are susceptible to damage from exposure to extreme temperatures. Mass deployment will require achieving true cold chain independence by increasing the sensor’s temperature stability. This is particularly important because many regions with endemic groundwater contamination concerns—for example, Kenya^25^, India^27^, Pakistan^28^, Bangladesh^29^, and others—have hot climates. One of the most promising avenues for increasing temperature stability is the addition of compounds called lyoprotectants that stabilize the system upon freeze-drying; some in vitro gene expression reactions can maintain integrity at 50 °C for up to a month when supplemented with appropriate lyoprotectants, though similar studies have not been performed in biosensing reactions^30^. Optimizing the lyophilization process for temperature stability and shelf life therefore stands to substantially improve the sensor’s robustness, ensuring accurate water quality data in the areas where it is most needed.

Furthermore, the continued inclusion of appropriate control reactions will be important for test accuracy. In addition to indicating test failure, control reactions are important for controlling for changes in reaction behavior caused by variation in ambient temperature. While changes in temperature would not affect the tests’ sensitivity or specificity, they would affect the reaction rate, and therefore time to detection. Other approaches can be used to improve accuracy, such as developing calibration approaches that can control for variability due to reaction inhibitors that may be present in some samples^31^.

There are several promising avenues to improve the usability of these tests. For one, shorter time to result would be less burdensome on participants, who were asked to look at the test color every hour. If issues with the ambiguity of color change arise in other settings, they could be resolved by using alternative colorimetric reporters and substrates^32^ to generate more vibrant outputs. Additionally, the development of purpose-built tools to rehydrate the freeze-dried tests and facilitate the interpretation of their results stands to substantially improve user experience. For example, the tests could be embodied in a lateral flow assay^33^, such as those used in at-home pregnancy tests, for greater clarity of interpretation. Future testing should also include test characterization in a wider variety of water sources, particularly acidic, alkaline, or mineral-rich samples that may inhibit the biological processes needed for sensor activation.

In sum, the ability for a biosensor test to correctly identify water contaminated with fluoride ≥1.5 ppm indicates enormous potential for a new approach to water quality diagnostics, one that requires far less equipment, expertize, infrastructure, and cost to operate. Indeed, the recent characterization of biological mechanisms to sense other priority contaminants including lead^34^, copper^35^, nitrites^36^, and arsenic^37^ suggest the possibility of analogous point-of-use tests^38^ for all of these analytes. The accuracy, simplicity, rapidity, relatively low cost, and field-friendliness of these tests would facilitate broad implementation, thereby democratizing knowledge about water safety for all.

## Methods

### Test manufacture

The DNA plasmid encoding the fluoride biosensor used in this study was assembled using Gibson assembly (New England Biolabs, Cat#E2611S) and purified using a Qiagen QIAfilter Midiprep Kit (QIAGEN, Cat#12143). Its coding sequence consists of the *crcB* fluoride riboswitch from *Bacillus cereus* regulating the production of the enzyme catechol 2,3-dioxygenase, all expressed under the constitutive *E. coli* sigma 70 consensus promoter J23119^39^. A complete sequence of the plasmid used is available on Addgene with accession number 128810 (pJBL7025) [https://www.addgene.org/128810/].

Cell-free biosensing reactions used in the tests were set up according to previously established protocols^20,40^. Briefly, reactions consist of cleared cellular extract, a reagent mix containing amino acids, buffering salts, crowding agents, enzymatic substrate, and an energy source, and a reaction-specific mix of template DNA and sodium fluoride in an approximately 30/30/40 ratio (Supplementary Table 3). Test reactions contained no sodium fluoride, while positive control reactions were supplemented with 1 mM sodium fluoride to induce gene expression. Template DNA concentration for both sets of reactions was 5 nM, determined by the maximal template concentration at which no color change was observed in the absence of fluoride.

During reaction setup, master mixes of cellular extract, reagent mix, and template mix were prepared for both test and positive control reactions in 1.7 mL microcentrifuge tubes. Individual reactions were then aliquoted into 20 µL volumes in PCR tube strips for lyophilization. After aliquoting on ice, PCR tube caps were pierced with a pin, strips were wrapped in aluminum foil, then the wrapped strips were immersed in liquid nitrogen for freeze-drying for approximately 3 min. Reactions were immediately transferred to a Labconco FreeZone 2.5 Liter −84 °C Benchtop Freeze-Dryer (Cat# 710201000) with a condenser temperature of −84 °C and pressure of 0.04 mbar and freeze-dried overnight (≥16 h).

After freeze-drying, tests were vacuum sealed (KOIOS Vacuum Sealer Machine, Amazon, Amazon Standard Identification Number (ASIN) B07FM3J6JF) in a food saver bag (KOIS Vacuum Sealer Bag, Amazon, ASIN B075KKWFYN), along with a desiccant (Dri-Card Desiccants, Uline, Cat# S-19582) (Supplementary Fig. 3). Vacuum sealed reactions were then paced in a light-protective outer bag (Mylar open-ended food bags, Uline, Cat# S-11661) and impulse heat-sealed (Metronic 8-inch Impulse Bag Sealer, Amazon, ASIN B06XC76JVZ) before shipping. Tests were also shipped with single-use 20 µL micropipettes (MICROSAFE® 20 µL, Safe-Tec LLC, Cat# 1020) for field operation.

### Test-kit shipment to Nakuru County, Kenya

A first shipment of biosensor tests was used to assess 33 water samples from the first 16 households surveyed. All of these tests resulted in a faint yellow color, regardless of water source or fluoride concentration established via fluorimeter. This was likely caused by thermal degradation of the tests during shipment with the commercial shipping agency. While previous studies report shelf stability for up to a year^20,41^, these figures were derived from storage in temperature-controlled laboratory conditions. Commercial shipment routes from Illinois, USA to Nairobi, Kenya pass through extremely hot regions, e.g., Dubai for this particular shipment. These conditions were much different from those in the previous study usability study in Costa Rica in which tests were transported by commercial air, with gentler shipping and storage conditions^20^. A laboratory investigation of test temperature stability indicated that elevated storage temperatures can indeed cause test components to degrade, resulting in a faint yellow color upon rehydration consistent with field observations (Supplementary Fig. 2).

The next batch of tests was therefore shipped refrigerated on January 25th, 2022, which we hypothesized would extend the tests’ shelf stability to align with earlier findings. After the tests were made and packaged, they were placed in a polystyrene foam-lined container before being covered with a NanoCool refrigeration system (Peli BioThermal). The container was then sealed shut and shipped using a standard commercial shipping service. This batch of tests was held in customs, refrigerated, until release on February 28th, 2022. These tests were used in the field from March 5th to March 14th, 2022 to generate the data on test accuracy reported in this manuscript.

As discoloration due to thermal degradation could confound the intended yellow hue in the presence of fluoride (i.e., false positives), we assessed test accuracy using only tests that had been refrigerated during shipping and transport to participants’ houses. The 33 water samples from the first 16 households were therefore excluded from analysis of test accuracy.

### Participant recruitment

Participants were recruited from six sublocations (Kelelwet, Kipsimbol, Kigonor, Parkview, Lalwet, and Mwariki) in Barut Ward within Nakuru County (Supplementary Fig. 4, geographic information adapted from OpenStreetMap^42^). This location was chosen because of high fluoride levels and familiarity with the communities by the study team.

Before any data were collected, community meetings were held in each sub-location to discuss study goals and objectives. After obtaining permission from the community and village assistant chiefs to conduct research, local community mobilizers were engaged to assist with identifying households eligible for participation. Individuals who were 18 years or older, had lived in Nakuru country for more than three months, relied on local water sources for drinking, had a child in the household, were willing to discuss their household water situation, and provide a sample of each source of water in the household for fluoride testing were eligible. We sought to recruit 10–12 participants from each of the five sublocations to ensure a range of sociodemographic characteristics and drinking water sources. Having a child resident was a criterion in order to elucidate community understandings about fluorosis in children.

### Data collection

After obtaining informed written consent, participants participated in a 30-min survey (cf. Supplementary Fig. 1 for a graphical overview of data collection). Topics included household sociodemographic information, knowledge, attitudes, and behaviors about fluoride and fluorosis, and household water insecurity using the validated Household Water Insecurity Experiences (HWISE) scale^43^. The 12 HWISE items query the frequency of experiences with water insecurity in the prior month; “never” is scored 0, “often/always” scored 3, for a range of 0–36. These data were collected to be able to investigate if user experiences or attitudes about testing varied by experiences with fluorosis or water insecurity. Participants were also asked about the number of sources of their water and willingness to provide and test water samples. Survey responses were recorded on tablets using Open Data Kit (ODK)^44^.

After completion of the survey, participants provided 1–3 samples of water from different household sources. They then received a brief (~5 min) explanation of the testing process, and then tested their own household samples using the fluoride biosensor tests. Each test consisted of a microtube that was a positive control, and a second microtube in which the sample of interest was tested. To test their samples, participants first removed the tests from the light-protective foil pouch and vacuum sealed pouch containing desiccant, both of which were then discarded (Supplementary Fig. 3). A micropipette was then filled with 20 µL water by slowly immersing it to the fill line. To dispense the water, the thumb and index finger were used to cover the holes in the micropipette while the bulb was squeezed with the other hand. The reactions were then incubated at ambient temperature for up to six hours, shorter if there was a visible color change. During this incubation time, participants were asked to check hourly for yellow color change and note the time taken for it to occur. Tests were expected to turn yellow if fluoride levels were ≥1.5 ppm, with no color change for tests of water below this level. All positive controls were expected to turn yellow. Color change was read after placing reactions against a white background for visual contrast.

The study team returned to conduct a second survey on user experiences with the testing process and to test the water samples using the gold-standard photometer within 6 h. Participants were asked about their experiences with the testing procedure as well as their interpretation of the color of the results of the sample and control tests. Photographs of the completed reactions were also taken at this time. Finally, quantitative fluoride measurements were taken by the field team with a Hanna Instruments Fluoride High Range Photometer Kit (Cat# HI97739C), a gold-standard method used to assess the accuracy of the bioengineered tests. Photometry results on actual measured fluoride concentrations of water samples were shared with and explained to participants. At the conclusion of the second survey, each participant was given KES 500 (USD 4.30) as remuneration for the time and effort spent participating in the research. Each participating household was also given a ceramic drinking water filter.

Data were collected from November 16th to November 23rd, 2021 and March 5th to March 14th, 2022. During surveying and water testing, participants and research assistants maintained COVID-19 protocols as per the local area guidelines. Study staff were vaccinated, maintained appropriate social distancing, sanitized hands, and cleaned field tools after each household visit.

### Data analysis

Data were exported from ODK into Microsoft Excel for analysis. Basic descriptive statistics were performed to describe participant socio-demographics and experiences with usability, including if participants’ interpretation of color change matched that of study staff. Open-ended items about fluoride and fluorosis knowledge, attitudes, and behavior were grouped thematically and coded independently by two authors. Knowledge-related responses were characterized as “correct” if consistent with conventional biomedical understanding, “incorrect”, or unfamiliar.

Tests were classified as ‘ON’ by the Kenya-based field team if they were visibly yellow after six hours, and ‘OFF’ if there was no observable color change by eye. These assessments were independently validated by the US-based team from photographs of the completed tests. Tests classified as ‘ON’ were marked true positive if they corresponded to a photometer measured fluoride concentration ≥1.5 ppm, and false positive if they corresponded to a photometer measured fluoride concentration <1.5 ppm. Tests classified as ‘OFF’ were marked as true negative if they corresponded to a photometer measured fluoride concentrations <1.5 ppm, and false positive if they corresponded to a photometer measured fluoride concentrations ≥1.5 ppm. Sensitivity was determined by the ratio of true-positive results to total-positive measurements (combined true and false positives), while specificity was determined by the ratio of true-negative results to total-negative measurements (combined true and false negatives), and calculated in Stata^45^. Confidence intervals for sensitivity and specificity were calculated using the diagt module in Stata using counts of true positives, true negatives, false positives, and false negatives.

Our target sample size for establishing test accuracy (Aim 1) was 65, based on the observed sensitivity of 0.93, and observed prevalence of 0.78^46^. Although we obtained 90 water samples, only 57 were suitable for this analysis (see “*Test Kit Shipment to Nakuru County, Kenya*”); robust estimates were still generated with this sample size. For usability testing (Aim 2), data on experiences with rehydration and interpretation from 36 individuals is well above the number recommended for usability studies^47,48^.

### Human subjects approval

We obtained ethical approval for this study from Northwestern University’s (IRB STU00215306) and Amref Health’s (AMREF-ESRC P1003/2021) Institutional Review Boards. We also received authorization from the Ministry of Planning and Development, Nakuru County, which is responsible for coordinating research activities in the county and relevant Ministries. All participants provided written consent to participate in the study activities, including consent to take pictures of the at-home testing. The authors affirm that human research participants provided informed consent for publication of the images in Fig. 3.

### Reporting summary

Further information on research design is available in the Nature Research Reporting Summary linked to this article.

## Supplementary information

Supplementary Material

Reporting Summary

## Data Availability

All source data for the main and SI figures were deposited open access in Northwestern’s Arch database (https://arch.library.northwestern.edu). Data can be accessed via 10.21985/n2-zyy5-cp15. A complete sequence of the plasmid used is available on Addgene with accession number 128810 (pJBL7025) [https://www.addgene.org/128810/].
