# Supplementary material for: The accuracy and usability of point-of-use fluoride biosensors in rural Kenya
Source: NPJ Clean Water. 2023 Feb 8;6(1):5. doi: 10.1038/s41545-023-00221-5 (PMC9905762; doi:10.1038/s41545-023-00221-5)
Supplement: Supplementary file 1 — Supplementary Material [file 41545_2023_221_MOESM1_ESM.pdf]

The accuracy and usability of point-of-use fluoride biosensors in rural Kenya

Walter Thavarajah[1,2,3,4]*, Patrick Mbullo Owuor[5,6,7]*, Diana Ross Awuor[8], Karlmax Kiprotich[9], Rahul Aggarwal[5], Julius B. Lucks[1,2,3,4#], and Sera L. Young[3,4,5,7#]

1 – Department of Chemical and Biological Engineering, Northwestern University, 2145 Sheridan Rd, Evanston, IL, 60208, USA
2 – Center for Synthetic Biology, Northwestern University, 2145 Sheridan Rd, Evanston, IL, 60208, USA
3 – Center for Water Research, Northwestern University, 2145 Sheridan Rd, Evanston, IL, 60208, USA
4 – Center for Engineering, Sustainability and Resilience, Northwestern University, 2145 Sheridan Rd, Evanston, IL, 60208, USA
5 – Department of Anthropology, Northwestern University, 1810 Hinman Avenue, Evanston, IL 60208, USA
6 – Institute for Policy Research, Northwestern University, 2040 Sheridan Road, Evanston IL 60208, USA
7 – Program of African Studies, Northwestern University, 620 Library Pl, Evanston, IL 60208, USA
8 – Department of Management Science and Project Planning, Nairobi University. P.O. BOX 30197, GPO, Nairobi, Kenya.
9 – Department of Epidemiology and Medical Statistics, School of Public Health, Moi University, P.O. Box 4606 - 30100, Eldoret, Kenya

* – These authors contributed equally to this work.
# – To whom correspondence should be addressed, jblucks@northwestern.edu, sera.young@northwestern.edu

Supplemental Table 1. Breakdown of operating costs for point-of-use fluoride biosensors and fluoride photometers.

Item	Vendor	Catalog Number	Cost per kit (USD)	Notes
Cell-Free Reaction	N/A	N/A	$0.56	3 tests, 3 positive controls
Catechol	Alfa Aesar	A10164	$0	Price is a fraction of a cent
PCR Tubes	BrandTech Scientific	781332	$0.48	6 tubes
20 µL micropipette	Safe-Tec LLC	1020	$0.90	6 pipettes
Dessicant	Uline	S-19581	$0.06	1 card
Vacuum bag	Amazon	B075KKWFYN	$0.09	6 inches
Light-protectant bag	Uline	S-11661	$0.09	1 bag
Biosensor total cost (per test kit)			$2.18	3 tests, 3 positive controls/kit
**Biosensor total cost (per sample)**			**$0.73**	1 test, 1 positive control
Photometer Reagents	Hanna Instruments	HI93739-03	$266	300 samples
**Photometer total cost (per sample)**			**$0.89**	

Supplemental Table 2. Sources and test results for water tests used to determine point-of-use fluoride biosensor accuracy (n=57). Red highlighting indicates where positive controls failed, or samples were classified as false positive or negative.

Sample Number	Water Source	Sample Treated?	Positive Control On?	Fluoride (ppm)	Test On?	Time to Result	Test Result
**107-1**	Rainwater Collection	No	Yes	0	No	No color change	True Negative
**107-2**	Mixed Rain/Borehole	No	Yes	5.4	Yes	3-4h	True Positive
**107-3**	Borehole	No	Yes	6.5	Yes	2h	True Positive
**108-1**	Rainwater Collection	No	Yes	0	No	No color change	True Negative
**108-2**	Borehole	No	Yes	6.5	Yes	4-5h	True Positive
**109-1**	Borehole	No	Yes	5.7	Yes	5h	True Positive
**110-1**	Borehole	No	Yes	6.6	Yes	1-2h	True Positive
**110-2**	Borehole	No	Yes	4.9	Yes	1-2h	True Positive
**111-1**	Borehole	No	Yes	5.8	Yes	2-3h	True Positive
**112-1**	Borehole	No	Yes	5.6	Yes	2-3h	True Positive
**113-1**	Rainwater Collection	No	Yes	0	No	No color change	True Negative
**113-2**	Mixed Rain/Borehole	No	Yes	7.9	Yes	2-3h	True Positive
**114-1**	Borehole	No	Yes	6.2	Yes	1-2h	True Positive
**114-2**	Borehole	No	Yes	5.5	Yes	3-4h	True Positive
**114-3**	Mixed Rain/Borehole	No	No	5.3	Yes	5h	True Positive
**115-1**	Borehole	No	Yes	6.1	Yes	2-3h	True Positive
**116-1**	Borehole	No	Yes	6	Yes	4-5h	True Positive
**117-1**	Mixed Rain/Borehole	No	Yes	3.9	Yes	3-4h	True Positive
**118-1**	Protected Dug Well	No	Yes	8.8	Yes	1-2h	True Positive
**118-2**	Borehole	Unknown	Yes	5.8	Yes	3-4h	True Positive
**119-1**	Rainwater Collection	No	No	0	Yes	5h	False Positive
**119-2**	Protected Dug Well	No	Yes	7.4	Yes	2-3h	True Positive
**120-1**	Borehole	Unknown	Yes	5.7	Yes	5h	True Positive
**121-1**	Borehole	No	Yes	5.8	Yes	3-4h	True Positive
**121-2**	Rainwater Collection	No	No	1.1	Yes	3-4h	False Positive
**122-1**	Borehole	No	No	6	Yes	3-4h	True Positive
**123-1**	Protected Dug Well	No	Yes	7.7	Yes	2-3h	True Positive
**124-1**	NAWASCO	Yes	Yes	5	Yes	3-4h	True Positive
**124-2**	Protected Dug Well	No	Yes	15.8	Yes	1-2h	True Positive

**125-1**	Protected Dug Well	No	Yes	18.7	Yes	3-4h	True Positive
**125-2**	Borehole	No	Yes	15.8	Yes	2-3h	True Positive
**126-1**	Rainwater Collection	No	Yes	0.4	No	No color change	True Negative
**126-2**	Protected Dug Well	No	Yes	17	Yes	1-2h	True Positive
**127-1**	Borehole	No	Yes	5.1	Yes	5h	True Positive
**128-1**	Borehole	No	Yes	5	Yes	3-4h	True Positive
**129-1**	Borehole	No	Yes	5.4	Yes	2-3h	True Positive
**130-1**	Rainwater Collection	No	Yes	0.4	No	No color change	True Negative
**130-2**	Borehole	No	Yes	7.2	Yes	2-3h	True Positive
**131-1**	Protected Dug Well	No	Yes	8.8	Yes	4-5h	True Positive
**132-1**	Rainwater Collection	No	Yes	0	Yes	5h	False Positive
**132-2**	Protected Dug Well	No	Yes	10.8	Yes	3-4h	True Positive
**133-1**	Rainwater Collection	Yes	Yes	0.5	No	No color change	True Negative
**133-2**	Protected Dug Well	No	Yes	8.8	Yes	5h	True Positive
**134-1**	Rainwater Collection	Yes	Yes	0.8	No	No color change	True Negative
**134-2**	Protected Dug Well	No	Yes	8.7	Yes	1-2h	True Positive
**135-1**	Surface Water	No	Yes	20	No	No color change	False Negative
**135-2**	Borehole	No	Yes	6	Yes	5h	True Positive
**136-1**	Borehole	No	Yes	6.3	Yes	5h	True Positive
**137-1**	Borehole	No	No	6.6	No	No color change	False Negative
**137-2**	Bagged Water	Yes	No	0.1	No	No color change	True Negative
**138-1**	Rainwater Collection	No	No	5.5	No	No color change	False Negative
**138-2**	Borehole	No	No	0.3	No	No color change	True Negative
**139-1**	Borehole	No	Yes	5.9	Yes	5h	True Positive
**139-2**	Mixed Rain/Borehole	No	No	5.8	Yes	3-4h	True Positive
**140-1**	Borehole	No	Yes	5.5	Yes	3-4h	True Positive
**141-1**	Borehole	No	Yes	6.4	Yes	2-3h	True Positive
**142-1**	Borehole	No	No	5.4	Yes	2-3h	True Positive

Supplemental Table 3. Biosensing reaction composition. Biosensors are assembled in a series of steps, outlined in the table below. The indicated volumes are for the assembly of ten test reactions and ten positive control reactions. Step 1: The reaction mixture is assembled from biochemical components and cellular extracts. The definitions of components and protocols for making each component can be found in Silverman *et al. ACS Synthetic Biology* (doi:10.1021/acssynbio.8b00430). Step 2: The DNA/inducer/water mixture is assembled for the two different types of reactions, test and positive control. Step 3: Reactions are assembled by mixing the reaction mixture from step 1 with the appropriate DNA/inducer/water mixture from Step 2, depending on the type of reaction. Once mixed, individual reactions are aliquoted into tubes in 20 μL aliquots. The procedure is designed to produce 10% more final mixture than needed to facilitate easier pipetting. A fillable reaction setup spreadsheet is available in Silverman *et al. ACS Synthetic Biology* (doi:10.1021/acssynbio.8b00430) if different numbers of reactions are desired, or the biosensor DNA template is at a different stock concentration.

**Step 1.** Assemble Reaction Mixture			
Component		Volume (μL)	
Salt solution		32.27	
NTP master mix		32.27	
Reagent mix		38.72	
Amino acids		19.36	
Energy mix		16.13	
Cell extract		145.20	
Total		283.95	
**Step 2.** Assemble DNA/Inducer/Water Mixture			
Reaction Type	Water (μL)	Biosensor DNA 530.20 nM Stock (μL)	Sodium Fluoride 50nM Stock (μL)
Test Reactions	51.10	39.83	0.00
Positive Control	8.86	39.83	42.24
**Step 3.** Assemble Reactions			
Component		Volume (μL)	
Reaction mixture (from Step 1)		129.07	
DNA/Inducer/Water mixture (from Step 2)		90.93	
Total		220.00	

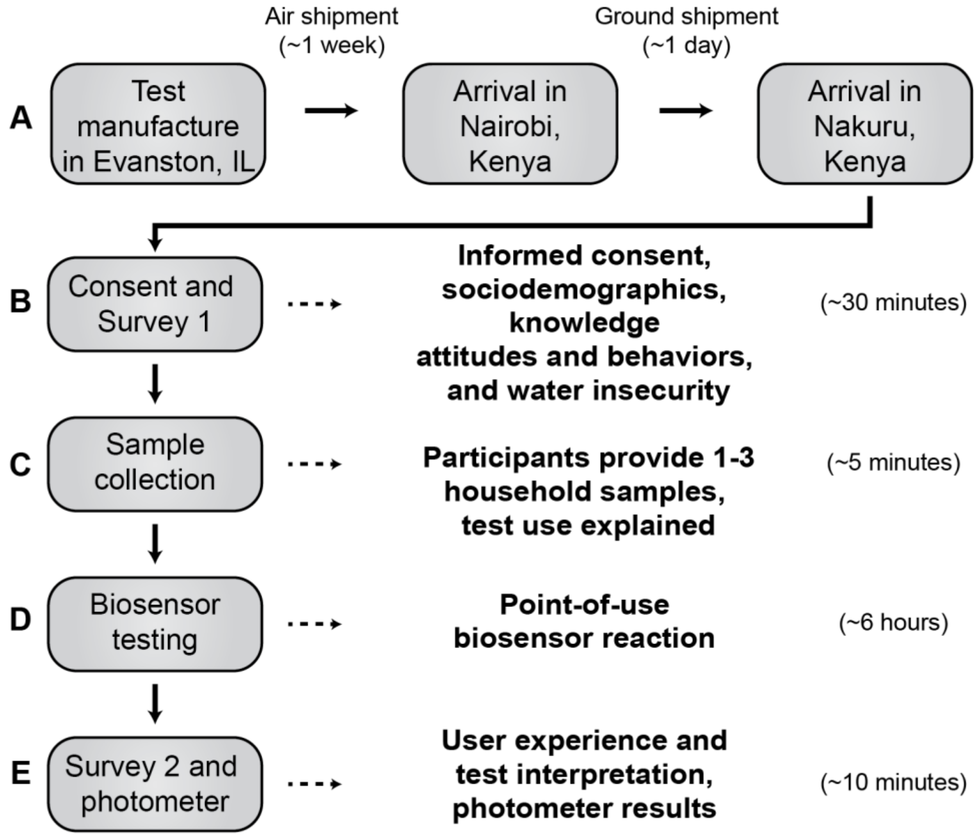

Supplementary Figure 1. Graphical workflow of the data collection process. Prior to the biosensors arriving in Kenya, members of the research team met with community leaders and mapped out villages and households for data collection. (A) Tests were manufactured in Evanston, Illinois, and then shipped to Nairobi, Kenya, before being transported to Nakuru County using a portable cooler box. Data collection in Barut Ward commenced immediately thereafter. (B) With the help of community leaders, study staff visited the participants households and obtained informed consent. This was then followed by Survey 1, which included questions about socio-demographics; knowledge, attitudes, and behaviors about fluoride and fluorosis; and water insecurity. Informed consent and Survey 1 took approximately 30 minutes. (C) Field staff then explained how to use the biosensor tests. Participants were asked to collect water samples from up to three sources in their households and then place drops of water from each source into separate biosensor tests. (D) Field staff typically left participants' houses while the biosensor was reacting, which could take up to six hours. Participants were asked to look at the color of the reaction every hour. (E) Field staff then returned to participants' homes for Survey 2, which was a 10-minute survey about experiences with the biosensor tests, including test output interpretation. Enumerators then tested each household water sample with the portable fluorimeter and shared the gold standard test results with the participant. At the conclusion of the study, participants received a water filter and 5 USD in appreciation for their time and effort.

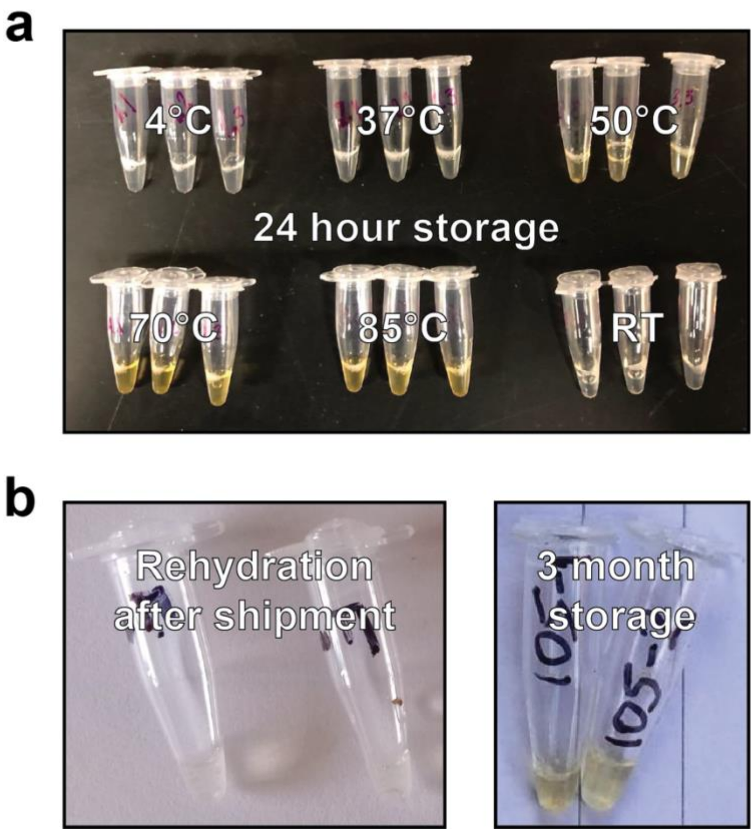

Supplemental Figure 2. Cell-free reactions degrade after storage at high temperatures. (a) Lyophilized reactions rehydrated with purified water after 24-hour storage at 4°C, 37°C, 50°C, 70°C, 85°C, and room temperature (RT, ~20°C). As temperatures increased, rehydration resulted in a yellow color due to component degradation and thus loss of function. This yellow color change confounds the interpretation of the intended yellow color change of the tests in the presence of fluoride causing false positives. (b) Tests from the first batch shipped to Kenya rehydrated immediately after arrival (left) and after three-month storage at ambient temperatures (right). Color change in the stored reactions resembles color change from 24-hour storage at high temperatures and was similarly reflective of loss of function.

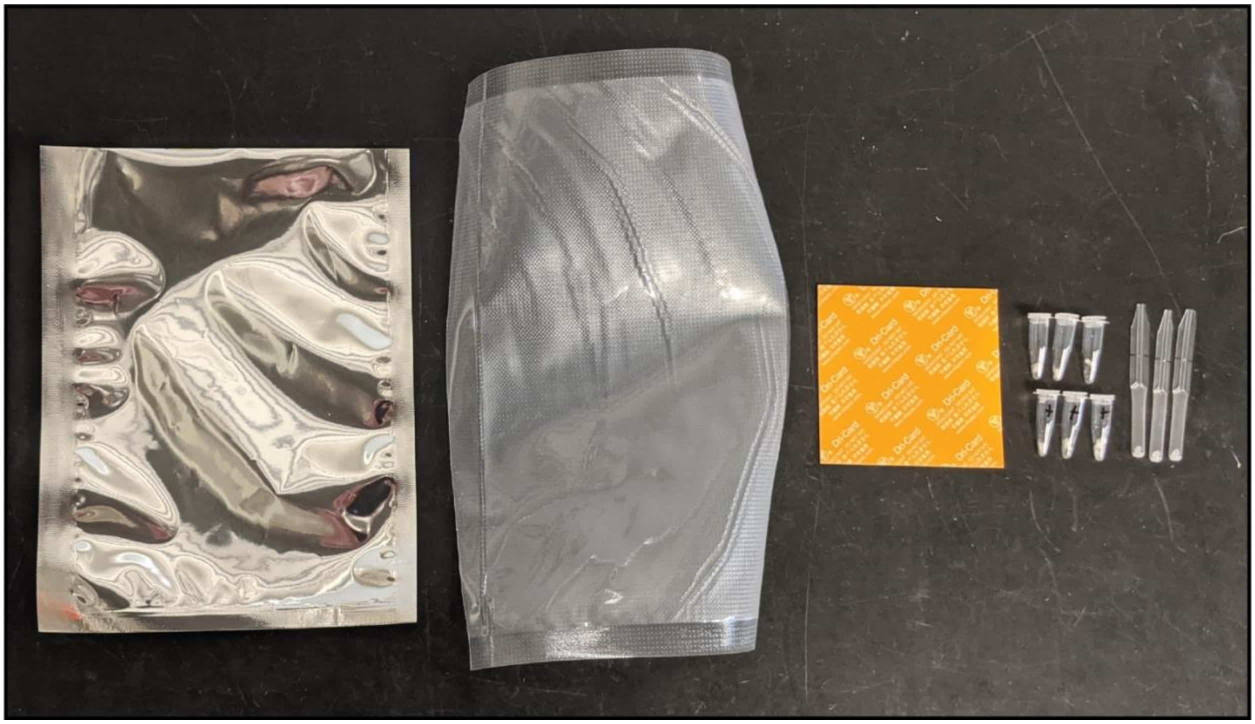

Supplemental Figure 3. Fluoride testing field kit. Pictured from left to right: foil pouch, vacuum bag, desiccant card, freeze-dried reactions in PCR tubes, and disposable micropipettes. Reactions and desiccant card are placed in the vacuum bag and sealed, then stored in the foil pouch to prevent photodegradation. Pipettes were packaged separately during testing but could also be stored in the foil pouch.

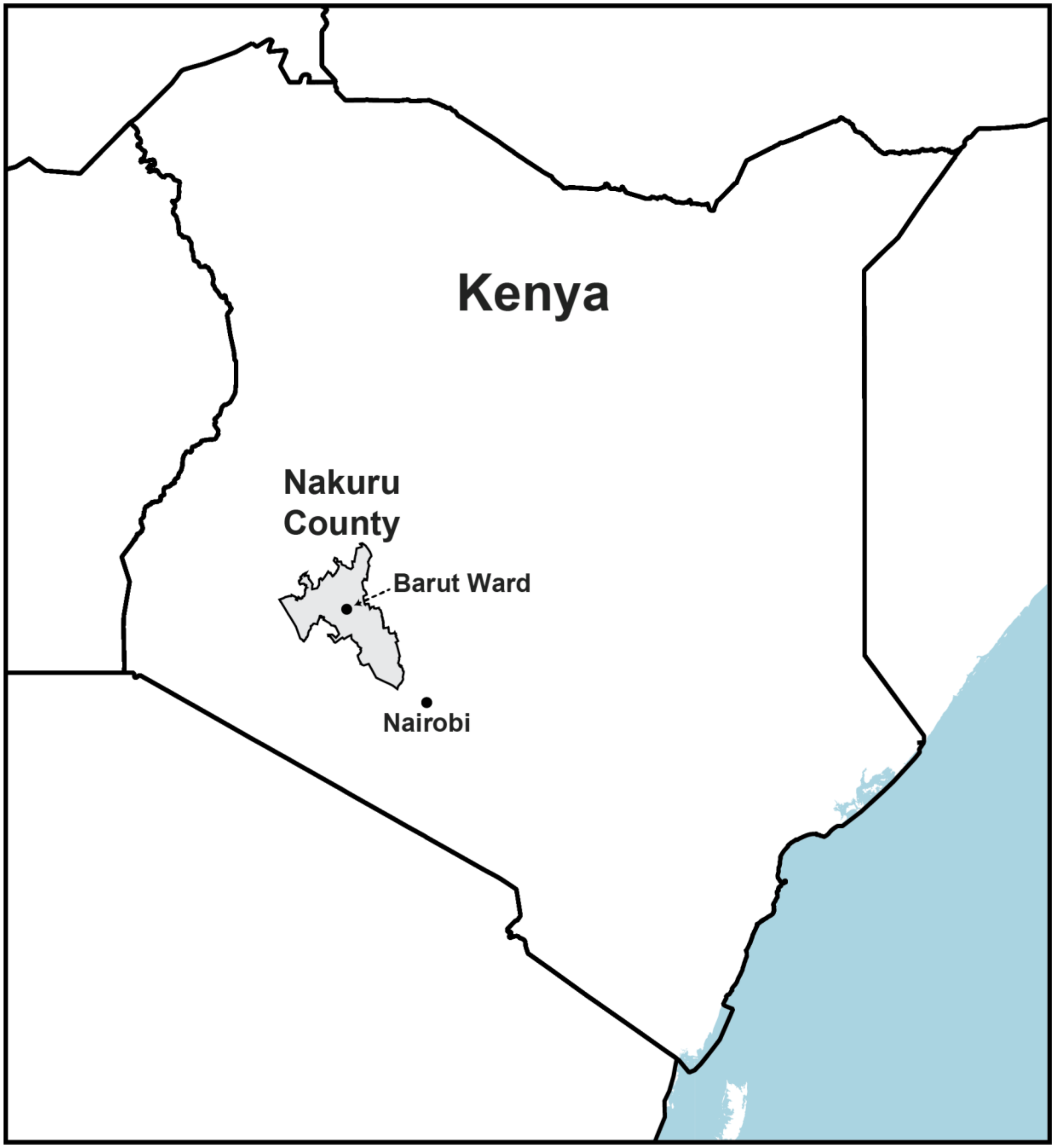

Supplemental Figure 4. Geographic location of the study site. Samples were collected in Barut Ward, one of 55 wards in Nakuru County. Nakuru County is located in the Great Rift Valley, where geogenic fluoride is common. Geographical data © OpenStreetMap contributors.
