# Supplementary material for: The accuracy and usability of point-of-use fluoride biosensors in rural Kenya
Source: NPJ Clean Water. 2023 Feb 8;6(1):5. doi: 10.1038/s41545-023-00221-5 (PMC9905762; doi:10.1038/s41545-023-00221-5)
Supplement: Supplementary file 2 — Reporting Summary [file 41545_2023_221_MOESM2_ESM.pdf]

nature portfolio

Corresponding author(s): Sera Young & Julius Lucks

Last updated by author(s): July 8, 2022

Reporting Summary

Nature Portfolio wishes to improve the reproducibility of the work that we publish. This form provides structure for consistency and transparency in reporting. For further information on Nature Portfolio policies, see our Editorial Policies and the Editorial Policy Checklist.

Statistics

For all statistical analyses, confirm that the following items are present in the figure legend, table legend, main text, or Methods section.

n/a	Confirmed	
☐	☒	The exact sample size (*n*) for each experimental group/condition, given as a discrete number and unit of measurement
☐	☒	A statement on whether measurements were taken from distinct samples or whether the same sample was measured repeatedly
☐	☒	The statistical test(s) used AND whether they are one- or two-sided<br>*Only common tests should be described solely by name; describe more complex techniques in the Methods section.*
☐	☒	A description of all covariates tested
☒	☐	A description of any assumptions or corrections, such as tests of normality and adjustment for multiple comparisons
☐	☒	A full description of the statistical parameters including central tendency (e.g. means) or other basic estimates (e.g. regression coefficient) AND variation (e.g. standard deviation) or associated estimates of uncertainty (e.g. confidence intervals)
☒	☐	For null hypothesis testing, the test statistic (e.g. *F*, *t*, *r*) with confidence intervals, effect sizes, degrees of freedom and *P* value noted<br>*Give P values as exact values whenever suitable.*
☒	☐	For Bayesian analysis, information on the choice of priors and Markov chain Monte Carlo settings
☒	☐	For hierarchical and complex designs, identification of the appropriate level for tests and full reporting of outcomes
☒	☐	Estimates of effect sizes (e.g. Cohen's *d*, Pearson's *r*), indicating how they were calculated

Our web collection on statistics for biologists contains articles on many of the points above.

Software and code

Policy information about availability of computer code

Data collection	Computer code was not used to collect data.
Data analysis	Stata, Microsoft Excel

For manuscripts utilizing custom algorithms or software that are central to the research but not yet described in published literature, software must be made available to editors and reviewers. We strongly encourage code deposition in a community repository (e.g. GitHub). See the Nature Portfolio guidelines for submitting code & software for further information.

Data

Policy information about availability of data

All manuscripts must include a data availability statement. This statement should provide the following information, where applicable:

- Accession codes, unique identifiers, or web links for publicly available datasets
- A description of any restrictions on data availability
- For clinical datasets or third party data, please ensure that the statement adheres to our policy

All source data for the main and SI figures were deposited open access in Northwestern's Arch database (https://arch.library.northwestern.edu). Data can be accessed via DOI: 10.21985/n2-zyy5-cp15.

Human research participants

Policy information about studies involving human research participants and Sex and Gender in Research.

Reporting on sex and gender	Gender-disaggregated data are appropriately reported.
Population characteristics	These are described in Table 1.
Recruitment	Participants were recruited from five sub-locations (Kelelwet, Kipsimbol, Kigonor, Parkview, Lalwet, and Mwariki) in Barut Ward within Nakuru County (Supplemental Figure 3, geographic information adapted from OpenStreetMap37). This location was chosen because of high fluoride levels and familiarity with the communities. Individuals who were 18 years or older, had lived in Nakuru country for more than three months, relied on local water sources, had a child in the household, were willing to discuss their household water situation, and provide a sample of each source of water in the household for fluoride testing were eligible. We sought to recruit 10-12 participants from each of the five sublocations to ensure a range of socio-demographic characteristics and drinking water sources. Having a child resident was a criterion in order to elucidate community understandings about fluorosis in children. After obtaining permission from the community and village assistant chiefs to conduct research, local community mobilizers were engaged to assist with identifying households eligible for participation.
Ethics oversight	Before any data were collected, community meetings were held in each sub-location to discuss study goals and objectives. After obtaining permission from the community and village assistant chiefs to conduct research, local community mobilizers were engaged to assist with identifying households eligible for participation. We obtained ethical approval for this study from Northwestern University's (IRB STU00215306) and Amref Health's (AMREF-ESRC P1003/2021) Institutional Review Boards. We also received authorization from the Ministry of Planning and Development, Nakuru County, which is responsible for coordinating research activities in the county and relevant Ministries. All participants provided written consent to participate in the study activities, including consent to take pictures of the at-home testing.

Note that full information on the approval of the study protocol must also be provided in the manuscript.

Field-specific reporting

Please select the one below that is the best fit for your research. If you are not sure, read the appropriate sections before making your selection.

☐ Life sciences ☒ Behavioural & social sciences ☐ Ecological, evolutionary & environmental sciences

For a reference copy of the document with all sections, see nature.com/documents/nr-reporting-summary-flat.pdf

Behavioural & social sciences study design

All studies must disclose on these points even when the disclosure is negative.

Study description	A survey was conducted with participants before and after the use of a point-of-use fluoride biosensor test.
Research sample	Participants were recruited from five sub-locations (Kelelwet, Kipsimbol, Kigonor, Parkview, Lalwet, and Mwariki) in Barut Ward within Nakuru County, Kenya (Supplemental Figure 3, geographic information adapted from OpenStreetMap37). This location was chosen because of high fluoride levels and familiarity with the communities. We sought to recruit 10-12 participants from each of the five sublocations to ensure a range of socio-demographic characteristics and drinking water sources.<br><br>Individuals who were 18 years or older, had lived in Nakuru country for more than three months, relied on local water sources, had a child in the household, were willing to discuss their household water situation, and provide a sample of each source of water in the household for fluoride testing were eligible. Having a child resident was a criterion in order to elucidate community understandings about fluorosis in children.<br><br>The data are not representative. A total of 90 water samples were collected amongst 52 participants; 73.1% of whom were female (see Table 1).
Sampling strategy	Before any data were collected, community meetings were held in each sub-location to discuss study goals and objectives. After obtaining permission from the community and village assistant chiefs to conduct research, local community mobilizers were engaged to assist with identifying households eligible for participation.
Data collection	After obtaining informed written consent, participants were participated in a 30 minute survey. Topics included household sociodemographic information, knowledge, attitudes, and behaviors about fluoride and fluorosis, and household water insecurity using the validated Household Water Insecurity Experiences (HWISE) scale38. The 12 HWISE items query the frequency of experiences with water insecurity in the prior month; "never" is scored 0, "often/always" scored 3, for a range of 0-36. These data were collected to be able to investigate if user experiences or attitudes about testing varied by experiences with fluorosis or water insecurity. Participants were also asked about the number of sources of their water and willingness to provide and test water

samples. Survey responses were recorded on tablets using Open Data Kit (ODK)39.

After completion of the survey, participants provided 1-3 samples of water from different household sources. They then received a brief (5 minute) explanation of the testing process, and then tested their own household samples using the fluoride biosensor tests. Each test consisted of a microtube that was a positive control, and a second microtube in which the sample of interest was tested. To test their samples, participants first removed the tests from the light-protective foil pouch and vacuum sealed pouch containing desiccant, both of which were then discarded (Supplemental Figure 2). A micropipette was then filled with 20µL water by slowly immersing it to the fill line. To dispense the water, the thumb and index finger were used to cover the holes in the micropipette while the bulb was squeezed with the other hand. The reactions were then incubated at ambient temperature for up to six hours, shorter if there was a visible color change. During this incubation time, participants were asked to check hourly for yellow color change and note the time taken for it to occur. Tests were expected to turn yellow if fluoride levels were >1.5 ppm, with no color change for tests of water below this level. All positive controls were expected to turn yellow. Color change was read after placing reactions against a white background for visual contrast.

The study team returned to conduct a second survey on user experiences with the testing process and to test the water samples using the gold standard photometer within 6 hours. Participants were asked about their experiences with the testing procedure as well as their interpretation of the color of the results of the sample and control tests. Photographs of the completed reactions were also taken at this time. Finally, quantitative fluoride measurements were taken by the field team with a Hanna Instruments Fluoride High Range Photometer Kit (Cat# HI97739C), a gold standard method used to assess the accuracy of the bioengineered tests. Photometry results on actual measured fluoride concentrations of water samples were shared with and explained to participants.

Timing

Data were collected from November 16th to November 23rd, 2021 and March 5th to March 14th, 2022.

Data exclusions

A total of 90 water samples were collected from 52 participants. Socio-demographics and knowledge, attitudes, and behaviors pertaining to fluoride and experiences with water insecurity were available for all 52 participants. The sample size available for evaluating test accuracy (Aim 1) and interpretation (Aim 2) was 57 water samples provided from 36 households. The number of samples was reduced from 90 to 57 because shipping conditions for the first batch of tests caused test degradation, making them unsuitable to evaluate accuracy and usability (see “Characterization of biosensor accuracy with household water samples”).

Non-participation

None.

Randomization

N/A

Reporting for specific materials, systems and methods

We require information from authors about some types of materials, experimental systems and methods used in many studies. Here, indicate whether each material, system or method listed is relevant to your study. If you are not sure if a list item applies to your research, read the appropriate section before selecting a response.

Materials & experimental systems

n/a	Involved in the study
☒	☐ Antibodies
☒	☐ Eukaryotic cell lines
☒	☐ Palaeontology and archaeology
☒	☐ Animals and other organisms
☒	☐ Clinical data
☒	☐ Dual use research of concern

Methods

n/a	Involved in the study
☒	☐ ChIP-seq
☒	☐ Flow cytometry
☒	☐ MRI-based neuroimaging
